# NEDD8 promotes radioresistance via triggering autophagy formation and serves as a novel prognostic marker in oral squamous cell carcinoma

**DOI:** 10.1186/s12935-023-02883-0

**Published:** 2023-03-08

**Authors:** Tsu-Zong Yuan, Hui-Yu Lin, Chia-Hao Kuei, Che-Hsuan Lin, Hsun-Hua Lee, Hsin-Lun Lee, Hsiao-Wei Lu, Chia-Yi Su, Hui-Wen Chiu, Yuan-Feng Lin

**Affiliations:** 1 Department of Radiation Oncology, Yuan’s General Hospital, Kaohsiung, 802 Taiwan; 2 School of Medicine, Fu Jen Catholic University, New Taipei City, 242 Taiwan; 3 Department of Breast Surgery and General Surgery, Division of Surgery, Cardinal Tien Hospital, Xindian District, New Taipei City, 23148 Taiwan; 4 Department of Urology, Division of Surgery, Cardinal Tien Hospital, Xindian District, New Taipei City, 23148 Taiwan; 5 Department of Otolaryngology, School of Medicine, College of Medicine, Taipei Medical University, Taipei, 11031 Taiwan; 6 Department of Otolaryngology, Taipei Medical University Hospital, Taipei Medical University, Taipei, 11031 Taiwan; 7 Department of Neurology, Taipei Medical University Hospital, Taipei Medical University, Taipei, 11031 Taiwan; 8 Department of Neurology, School of Medicine, College of Medicine, Taipei Medical University, Taipei, 11031 Taiwan; 9 Department of Neurology, Vertigo and Balance Impairment Center, Shuang Ho Hospital, Taipei Medical University, New Taipei City, 23561 Taiwan; 10 Department of Radiology, School of Medicine, College of Medicine, Taipei Medical University, Taipei, 11031 Taiwan; 11 Department of Radiation Oncology, Taipei Medical University Hospital, Taipei, 11031 Taiwan; 12 Department of Otolaryngology Head and Neck Surgery, Shuang Ho Hospital, Taipei Medical University, New Taipei City, 23561 Taiwan; 13 Graduate Institute of Clinical Medicine, College of Medicine, Taipei Medical University, Taipei, 11031 Taiwan; 14 Department of Biomedical Engineering, Johns Hopkins University School of Medicine, Baltimore, MD USA; 15 Department of Medical Research, Shuang Ho Hospital, Taipei Medical University, New Taipei City, 23561 Taiwan; 16 TMU Research Center of Urology and Kidney, Taipei Medical University, Taipei, 11031 Taiwan; 17 Cell Physiology and Molecular Image Research Center, Wan Fang Hospital, Taipei Medical University, Taipei, 11696 Taiwan

## Abstract

**Background:**

Radiotherapy is the first-line regimen for treating oral squamous cell carcinoma (OSCC) in current clinics. However, the development of therapeutic resistance impacts the anticancer efficacy of irradiation in a subpopulation of OSCC patients. As a result, discovering a valuable biomarker to predict radiotherapeutic effectiveness and uncovering the molecular mechanism for radioresistance are clinical issues in OSCC.

**Methods:**

Three OSCC cohorts from The Cancer Genome Atlas (TCGA), GSE42743 dataset and Taipei Medical University Biobank were enrolled to examine the transcriptional levels and prognostic significance of neuronal precursor cell-expressed developmentally downregulated protein 8 (NEDD8). Gene set enrichment analysis (GSEA) was utilized to predict the critical pathways underlying radioresistance in OSCC. The colony-forming assay was used to estimate the consequences of irradiation sensitivity after the inhibition or activation of the NEDD8-autophagy axis in OSCC cells.

**Results:**

NEDD8 upregulation was extensively found in primary tumors compared to normal adjacent tissues and potentially served as a predictive marker for the therapeutic effectiveness of irradiation in OSCC patients. NEDD8 knockdown enhanced radiosensitivity but NEDD8 overexpression reduced it in OSCC cell lines. The inclusion of MLN4924, a pharmaceutical inhibitor for NEDD8-activating enzyme, dose-dependently restored the cellular sensitivity to irradiation treatment in irradiation-insensitive OSCC cells. Computational simulation by GSEA software and cell-based analyses revealed that NEDD8 upregulation suppresses Akt/mTOR activity to initiate autophagy formation and ultimately confers radioresistance to OSCC cells.

**Conclusion:**

These findings not only identify NEDD8 as a valuable biomarker to predict the efficacy of irradiation but also offer a novel strategy to overcome radioresistance via targeting NEDD8-mediated protein neddylation in OSCC.

**Supplementary Information:**

The online version contains supplementary material available at 10.1186/s12935-023-02883-0.

## Background

Oral squamous cell carcinoma (OSCC), which may originate in any of the tissues from the mouth with varied histologic types, constitutes a majority of head and neck squamous cell carcinoma (HNSCC). The 5-year survival rate is approximately 57% in newly diagnosed patients [1]. Betel quid chewing, alcohol consumption, and tobacco smoking have been documented as risk factors for oral cancer [2]. Tumor resection followed by adjuvant radiotherapy or chemoradiotherapy is the main therapeutic strategy for treating clinical OSCC patients [3, 4]. However, the development of radioresistance impacts the anticancer effectiveness of irradiation in the subpopulation of OSCC patients. As a result, uncovering the molecular mechanism to overcome radioresistance and identifying a useful biomarker to predict the effectiveness of radiotherapy against OSCC is urgently needed.

Neuronal precursor cell-expressed developmentally downregulated protein 8 (NEDD8) is a ubiquitin-like protein (UBL) that governs the posttranslational modification (PTM) pathway called neddylation [5]. Recent reports have demonstrated that NEDD8 promotes tumor progression and predicts a poor prognosis in patients with colorectal cancer [6], nasopharyngeal carcinoma [7] and bladder cancer [8]. Therefore, targeting NEDD8 activity has become a promising anticancer strategy [9]. Recently, a small-molecule pevonedistat, also named MLN4924, was developed to inhibit NEDD8-mediated neddylation and appeared to effectively suppress tumor growth in several cancer types [10–13]. Moreover, targeting NEDD8-related PTMs has also been reported to enhance cellular sensitivity to ionizing radiation in hormone-resistant prostate [14], pancreatic [15] and head and neck cancers [16]. Nevertheless, the effectiveness of targeting NEDD8 activity to enhance radiosensitivity and the molecular mechanism by which NEDD8 confers radioresistance in OSCC remains largely unknown.

To this end, this study explored the oncogenic role of NEDD8 in promoting radioresistance and estimated the effectiveness of targeting NEDD8-mediated PTM on reinforcing the anticancer efficacy of irradiation in OSCC. Our data showed that NEDD8 upregulation is extensively found in primary tumors compared to normal tissues and significantly correlates with a poorer prognosis in OSCC patients. NEDD8 expression was shown to negatively correlate with the therapeutic efficacy of irradiation in OSCC patients and a panel of OSCC cell lines. Whereas NEDD8 knockdown, as well as the inhibition of neddylation by MLN4924, dramatically enhanced irradiation responsiveness, NEDD8 overexpression predominantly reduced the cytotoxic effectiveness of irradiation on OSCC cells. Computational simulation using Gene Set Enrichment Analysis (GSEA) software indicated that NEDD8 upregulation is probably associated with inactivation of the PI3K/Akt/mTORC1 pathway, which is known to negatively regulate the formation of autophagosomes in OSCC. Moreover, our results demonstrated that MLN4924 treatment predominantly restores the responsiveness of OSCC cells to irradiation treatment by activating the PI3K/Akt/mTORC1 pathway and thereby suppressing autophagy formation. These findings provide a novel therapeutic strategy by targeting the NEDD8-mediated PTM to combat radioresistant OSCC.

## Materials and methods

### Data processing and sample collection

The pathological information and molecular data obtained by RNAseq of TCGA head and neck squamous cell carcinoma patients were collected from the UCSC Xena website (UCSC Xena. Available online: http://xena.ucsc.edu/welcome-to-ucsc-xena/). The raw intensities of mRNA levels derived from the GSE42743 dataset were downloaded from the Gene Expression Omnibus (GEO) database on the NCBI website and normalized by robust multichip analysis using GeneSpring GX11 (Agilent Technologies). The mRNA expression levels were normalized by the median of the detected samples and presented as log2 values. Paired normal tissues and primary tumors of OSCC patients were obtained from Taipei Medical University Biobank and collected in accordance with institutional review board approval (N202204007) and the Declaration of Helsinki.

### Cell culture

Oral cancer cell lines SAS, HSC2, HSC3 and HSC4 from the Japanese Collection of Research Bioresources (JCRB) Cell Bank were cultured in Dulbecco's modified Eagle's medium (DMEM) supplemented with 10% fetal bovine serum and 1% nonessential amino acids at 37 °C with 7% CO_2_.

### Colony formation assay

For clonogenic cell survival, cells (2 × 10^3^) treated with irradiation at designated dosages were grown on polystyrene 6-well plates. After 2 weeks of cultivation, the cells were fixed with 80% ethanol and then stained with 1% crystal violet. To quantify the relative colony formation, 30% acetic acid was used to solubilize the remaining crystal violet after several washes, and the optical density of solubilized crystal violet at 595 nm wavelength was determined by a photometer.

### Radiation exposure

Cells grown in 25 T flasks were exposed to 6 MV X-rays using a linear accelerator (Digital M Mevatron Accelerator, Siemens Medical Systems, CA, USA) at a dose rate of 8 Gy/min. A tissue-equivalent bolus (2 cm) was placed on top of the 25 T flasks to ensure electronic equilibrium. Tissue-equivalent material (10 cm) was placed under the flasks to obtain full backscatter.

### Plasmid construction

The genes encoding NEDD8 were amplified from human cDNA (Invitrogen) using the standard polymerase chain reaction (PCR) procedure with paired primers and subcloned into the lentiviral shuttle vector pLAS3w/puro. All lentiviral shRNA constructs were purchased from the National RNAi Core Facility Platform in Taiwan. The production of lentiviral particles used to establish stable cell lines was also performed in collaboration with the National RNAi Core Facility.

### Lentivirus-driven shRNA infection

Prior to lentiviral particle infection at a multiplicity of infection (MOI) of 2–10, cells with 50% confluence grown in 6-well plates were replenished with fresh medium containing 5 μg/ml polybrene (Santa Cruz). Stable clones were obtained after puromycin (10 μg/ml) selection. To validate the efficiency of NEDD8 knockdown and overexpression, cell lysates from puromycin-resistant cells were subjected to Western blot analysis.

### Reverse transcription PCR (RT‒PCR) and quantitative PCR (Q-PCR)

A TRIzol extraction kit (Invitrogen) was employed to extract total RNA from the detected cells. Aliquots (5 μg) of total RNA were converted to cDNA by M-MLV reverse transcriptase (Invitrogen) and then amplified by PCR with Taq-polymerase (Protech) using paired primers (for NEDD8, forward- CCGGAAAGGAGATTGAGATTGAC and reverse- CAACACCAGGTGAAGGACTGAAC; for GAPDH, forward-AGGTCGGAGTCAACGGATTTG and reverse-GTGATGGCATGGACTGTGGTC). qPCR was performed by using Power SYBRTM Green PCR Master Mix (Thermo Fisher). The obtained mRNA level of the detected gene was further normalized to the mRNA level of GAPDH. The 2^−ΔΔCt^ method was used to calculate the fold changes.

### Western blotting assay

Aliquots of cell lysate (20–100 μg) were loaded into each well of an SDS gel, separated by electrophoresis and then transferred to PVDF membranes. Prior to incubation with primary antibodies against phosphorylated Akt (Thr308), Akt, phosphorylated mTOR, mTOR, ATG5, Beclin-1, LC3-I/II (Cell Signaling) or GAPDH (AbFrontier) overnight at 4 °C, the membranes were incubated with blocking buffer (5% skim milk in TBS containing 0.1% Tween-20) for 2 h at room temperature. After several washes, the membranes were further incubated with a peroxidase-labeled secondary antibody for another 1 h at room temperature. Immunoreactive bands were finally visualized by using an enhanced chemiluminescence system (Amersham Biosciences, Tokyo, Japan). Uncut blots can be found in Additional file 1: Figs. S1 and S2.

### Statistical analysis

SPSS 17.0 software (Informer Technologies, Roseau, Dominica) was used to analyze statistical significance. A paired t test was utilized to compare NEDD8 gene expression in cancer tissues and corresponding normal tissues. Pearson’s and nonparametric Spearman’s correlation tests were performed to estimate the association among NEDD8 mRNA levels, clinical parameter values and other somatic gene expression levels in the detected primary tumors. Kaplan‒Meier analysis and log-rank tests were used to evaluate survival probabilities. The statistical significance of NEDD8 expression in clinical samples was analyzed by Student’s t test and one-way ANOVA with Tukey’s test.

## Results

### NEDD8 upregulation is extensively detected in primary tumors compared to normal tissues and is related to a poorer prognosis in oral cancer

**Fig. 1:**
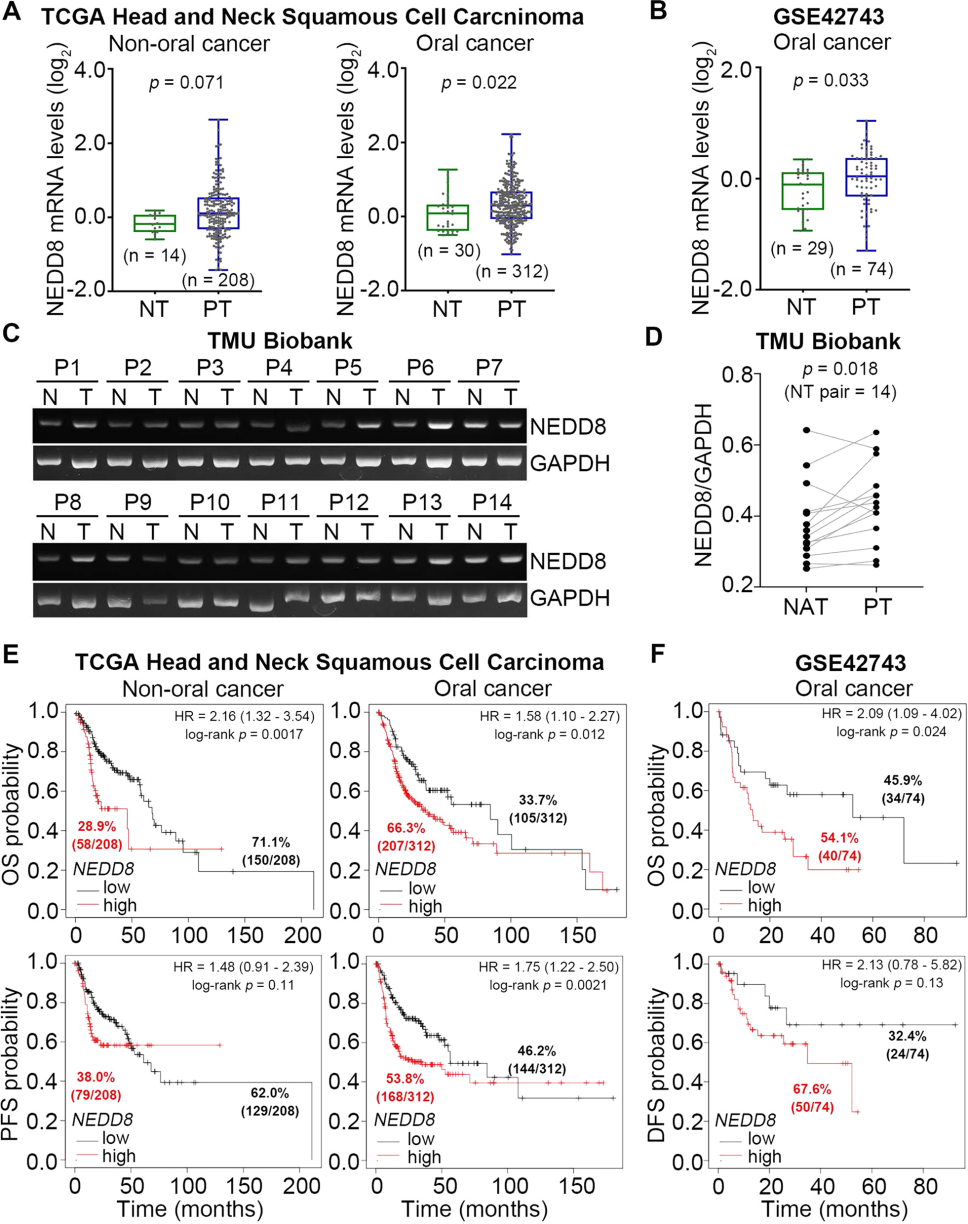
NEDD8 is upregulated and serves as a poor prognostic marker in OSCC. **A** and **B** Boxplots for the transcriptional profiling of NEDD8 in normal tissue (NT) and primary tumor (PT) derived from TCGA head and neck cancer patients divided into nonoral cancer and oral cancer subtypes (**A**) and GSE42743 oral cancer patients (**B**). Student’s t test was used to analyze the statistical significance. **C** and **D** RT‒PCR results for NEDD8 and GAPDH mRNA levels in paired normal (N) and tumor (T) tissues derived from 14 OSCC patients (P1–P14) and obtained from the Taipei Medical University (TMU) biobank (**C**). The NEDD8 mRNA levels normalized to the respective GAPDH controls in 14 paired normal adjacent tissue (NAT) and primary tumor (PT) specimens are shown in D. A paired t test was used to analyze the statistical significance. **E** and **F** Kaplan‒Meier analyses using overall survival (OS), progression-free survival (PFS) and disease-free survival (DFS) probability under minimized log-rank p values for NEDD8 mRNA levels against TCGA head and neck cancer patients with nonoral cancer or oral cancer subtypes (**E**) and GSE42743 oral cancer patients (**F**). The inserts denote the proportion of the enrolled patients in each group

Since oral cancer is a head and neck squamous cell carcinoma (HNSCC), we first delineated the association of NEDD8 expression with HNSCC tumorigenesis. By using The Cancer Genome Atlas (TCGA) HNSCC database, we performed transcriptional profiling of NEDD8 in normal tissues and primary tumors derived from nonoral and oral cancer patients (Fig. 1A). We found that the mRNA levels of NEDD8 in primary tumors were significantly (p = 0.022) higher than those in normal tissues from TCGA oral cancer patients but not nonoral cancer patients (Fig. 1A). A similar result was also found in the normal tissues and primary tumors of oral cancer patients from the GSE42743 dataset deposited in the Gene Expression Omnibus (GEO) database (Fig. 1B). Accordingly, RT‒PCR results showed that NEDD8 is highly expressed by primary tumors compared to normal adjacent tissues derived from the oral cancer biobank of Taipei Medical University (Fig. 1C and D).

Kaplan‒Meier analyses using overall and progression-free survival probability for NEDD8 mRNA levels showed that NEDD8 is a poor prognostic biomarker in TCGA nonoral cancer and oral cancer patients (Fig. 1E). Of note, under minimized log-rank p values, we found that the majority (66.3%) of TCGA oral cancer patients, but not nonoral cancer patients, harbored higher NEDD8 expression and had shorter overall and progression-free survival times (Fig. 1E). This finding was further validated by another Kaplan‒Meier analysis against GSE42743 oral cancer patients under overall and disease-free survival conditions (Fig. 1F). Importantly, the Cox repression test using overall survival conditions against GSE42743 oral cancer patients indicated that NEDD8 expression compared to other clinical confounders, such as age, sex, pathologic T/N stage, extracapsular spread (ECS), smoking and AJCC4 classification, was an independent prognostic biomarker (Table 1).

**Table 1:** Cox univariate and multivariate analyses under the condition of overall survival probability against *NEDD8* mRNA expression levels and clinical confounders derived GSE42743 cohort with OSCC

Overall survival (n = 74)				
Variables	Crude HR (95% CI)	*P*	Adjusted HR (95% CI)	*P*
Age				
< 60	1	NA	1	NA
≥ 60	1.52 (0.82–2.79)	0.183	1.33 (0.65–2.70)	0.438
Gender				
Female	1	NA	1	NA
Male	0.68 (0.34–1.36)	0.270	0.73 (0.34–1.57)	0.424
pT				
T1-T2	1	NA	1	NA
T3-T4	1.75 (0.90–3.41)	0.100	0.97 (0.40–2.34)	0.943
pN				
N0	1	NA	1	NA
N1-N2C	3.67 (1.73–7.80)	0.001	3.88 (1.07–14.0)	0.039
Ecs				
No	1	NA	1	NA
Yes	1.98 (1.07–3.69)	0.030	0.74 (0.34–1.63)	0.458
Smoker				
Yes	1	NA	1	NA
No	1.14 (0.54–2.40)	0.734	1.11 (0.48–2.57)	0.809
ajcc4				
1–2	1	NA	1	NA
3–4	2.91 (1.21–7.02)	0.017	1.17 (0.23–6.06)	0.854
*NEDD8* levels				
Low	1	NA	1	NA
High	2.09 (1.03–4.02)	0.027	2.06 (1.03–4.13)	0.041

### NEDD8 upregulation correlates with poor irradiation responsiveness in oral cancer

**Fig. 2:**
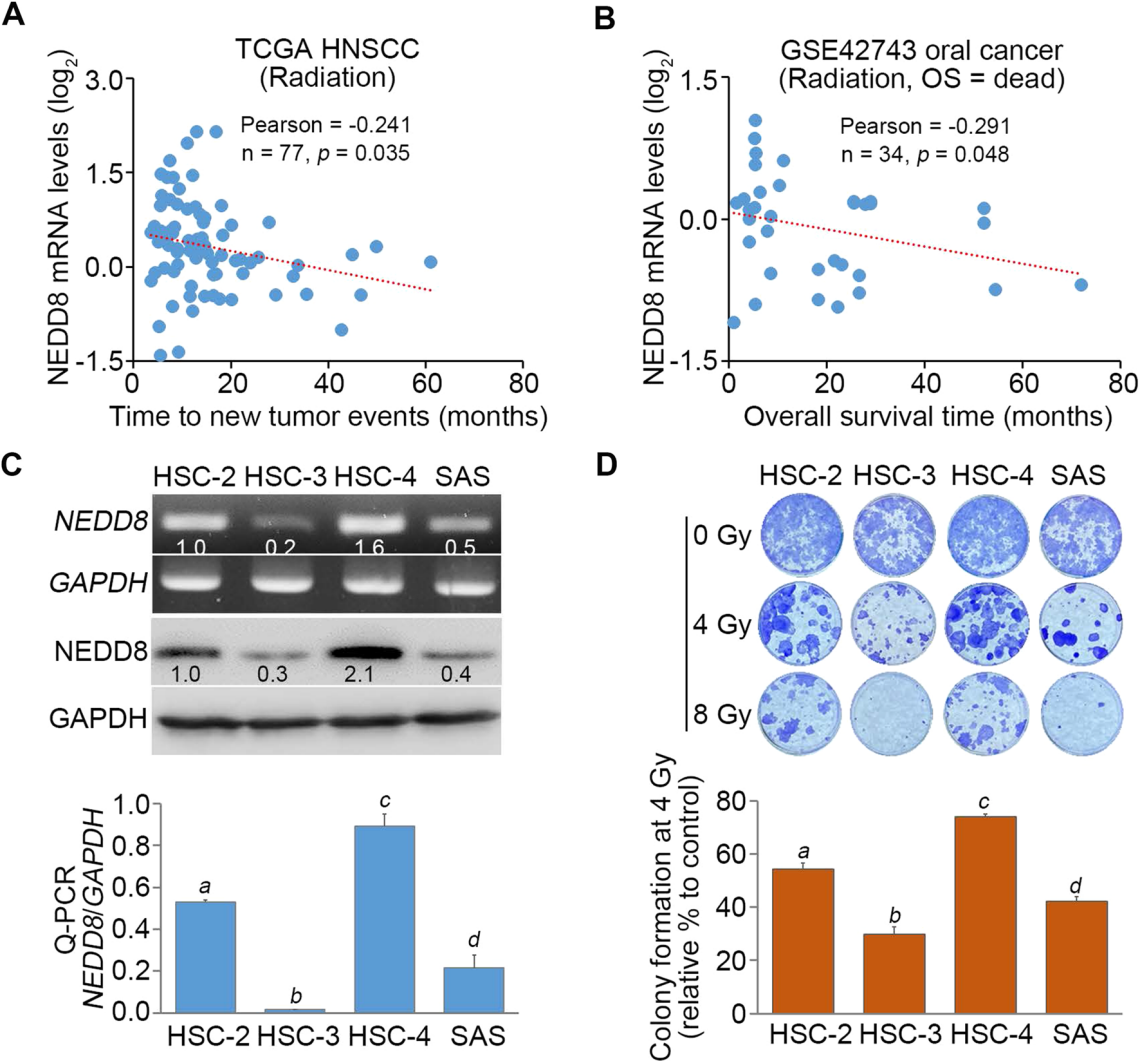
NEDD8 upregulation predicts a poor response to radiotherapy in oral cancer. **A** and **B** Dot plots for the correlation of NEDD8 mRNA levels with time to new tumor events (**A**) and overall survival time (**B**) in the radiation-treated patients derived from the TCGA HNSCC database and GSE42743 dataset, respectively. The Pearson correlation test was used to analyze the statistical significance. **C** The mRNA levels by RT‒PCR/Q-PCR and protein levels determined by Western blotting for NEDD8 detected in a panel of oral cancer cell lines HSC-2, HSC-3, HSC-4 and SAS. **D** Representative crystal violet staining for the colony formation of HSC-2, HSC-3, HSC-4 and SAS cells treated with the indicated irradiation dosages at the end of experiments (upper). The differences in colony formation post exposure to 4 Gy irradiation in the detected OSCC cells are shown in the histogram (lower). In **C** and **D**, the error bars denote the data from three independent experiments presented as the mean ± SEM, and the different letters indicate the statistical significance at p < 0.001 estimated by one-way ANOVA using Tukey’s post hoc tests

To understand the correlation of NEDD8 expression with radiosensitivity in oral cancer patients, we performed Pearson’s correlation tests for NEDD8 expression and time to new tumor event or overall survival time in oral cancer patients who were recorded to receive radiotherapy. The data showed that NEDD8 mRNA levels significantly (p < 0.05) and negatively correlated with time to new tumor event in the TCGA HNSCC cohort with irradiation treatment (Fig. 2A) and overall survival time in GSE42743 oral cancer patients receiving radiotherapy (Fig. 2B).

We next examined the association between NEDD8 expression and radiosensitivity in a panel of oral cancer cell lines. The data showed that the mRNA levels detected by RT‒PCR/quantitative PCR and the protein levels determined by Western blot analysis appeared to be relatively lower in HSC-3 cells and abundantly expressed by HSC-4 cells (Fig. 2C). Moreover, the cell viability postexposure to 4 Gy irradiation was dramatically reduced in HSC-3 cells but relatively higher in HSC-4 cells (Fig. 2D). The correlation between NEDD8 expression and cell viability postexposure to 4 Gy irradiation in the detected oral cancer cell lines was positively correlated.

**Fig. 3:**
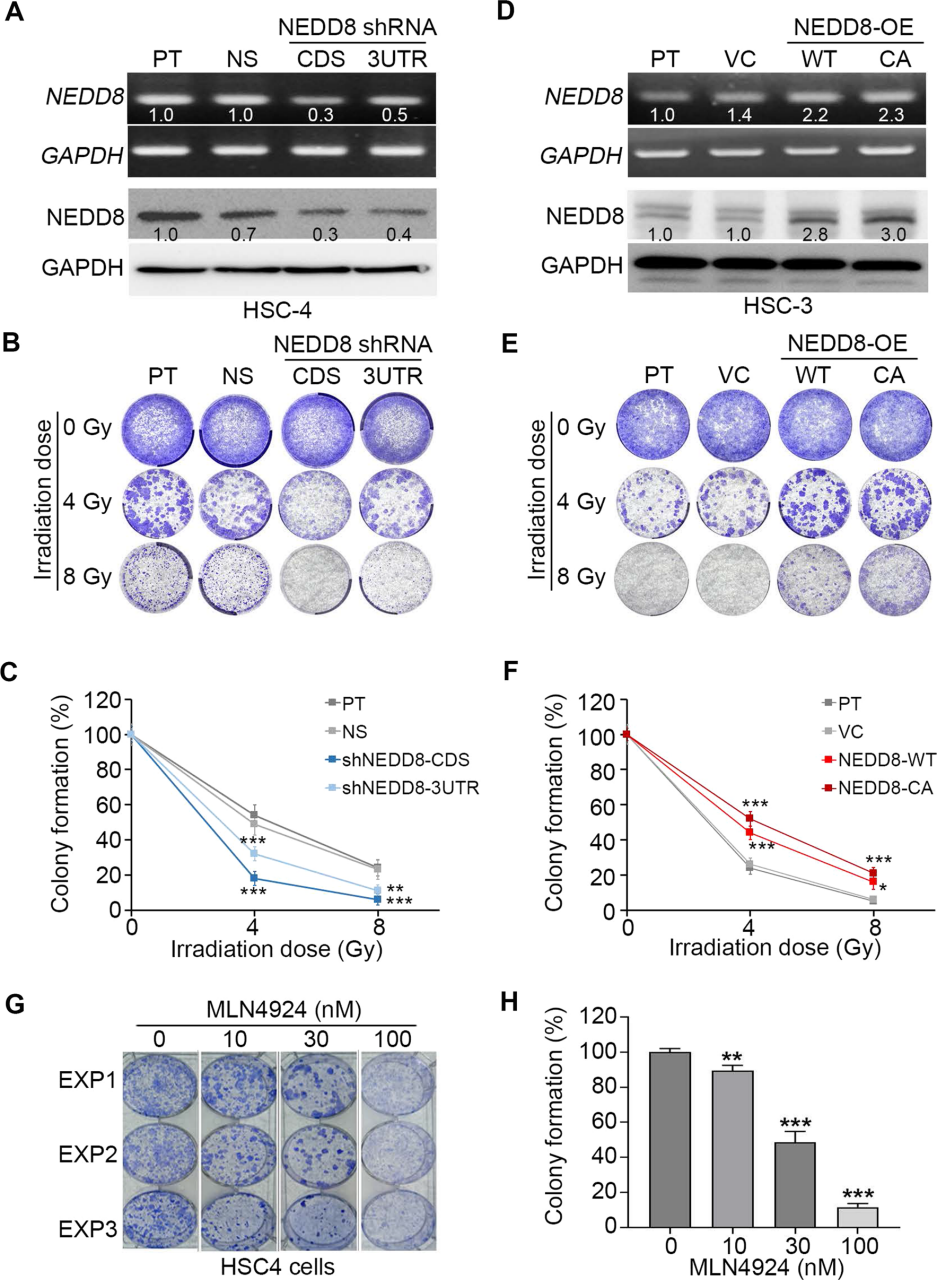
NEDD8 expression alters the radiosensitivity of oral cancer cells. **A** RT‒PCR (upper) and Western blot (lower) analyses of NEDD8 mRNA and protein levels, respectively, in the parental (PT) HSC4 cells and HSC4 cells transfected with nonsilencing (NS) control and 2 independent NEDD8 shRNAs. **B** and **C** Representative crystal violet staining (**B**) and the results obtained from three independent colony formation assays (**C**) for the indicated HSC4 cell variants postexposure to the designed irradiation doses. **D** RT‒PCR (upper) and Western blot (lower) analyses of NEDD8 mRNA and protein levels, respectively, in parental HSC3 cells and HSC3 cells transfected with vector control (VC) and plasmid-containing wild-type or constitutively active (CA) NEDD8 gene. **E** and **F** Representative crystal violet staining (**E**) and data from three independent colony formation assays (**F**) for the indicated HSC3 cell variants after treatment with the designed irradiation doses. **G** and **H** Crystal violet staining (**G**) and histogram (**H**) for data from three independent experiments (EXPs) of colony formation assay for HSC4 cells pretreated with the indicated concentration of MLN4924 for 24 h followed by irradiation exposure at 4 Gy. The symbols “*”, “**” and “***” represent statistical significance at p < 0.05, 0.01 and 0.001, respectively

To delineate whether NEDD8 upregulation is associated with a poor response to irradiation treatment, we further performed gene knockdown in HSC-4 cells, which exhibited a higher NEDD8 level and poor irradiation sensitivity (Fig. 3A). In comparison with parental and nonsilencing control cells, NEDD8 knockdown by 2 independent shRNA clones with the target DNA sequence corresponding to the coding DNA sequence (CDS) and 3’-untranslated region (3UTR) of the NEDD8 gene dramatically reduced the endogenous mRNA and protein levels of NEDD8 (Fig. 3A) and significantly (p < 0.01) restored the cellular irradiation sensitivity (Fig. 3B and C). Conversely, the enforced expression of exogenous wild-type (WT) and constitutively active (CA) constructs of the NEDD8 gene predominantly enhanced the mRNA and protein levels of NEDD8 (Fig. 3D) and rendered HSC-3 cells express a lower NEDD8 level, are relatively radiosensitive, and more resistant to irradiation treatment (Fig. 3E and F). Notably, HSC3 cells overexpressing constitutively active NEDD8 showed a poorer irradiation response than wild-type NEDD8-overexpressing HSC3 cell variants (Fig. 3E and F), implying the involvement of its enzymatic modification, neddylation, toward target proteins in radioresistance in oral cancer. We further validated these findings by pretreating HSC4 cells with MLN4924, a specific inhibitor of NEDD8-activating enzyme, before irradiation exposure. The data revealed that MLN4924 dose-dependently enhanced the cytotoxic effectiveness of irradiation on HSC4 cells (Fig. 3G and H).

### NEDD8 expression/activity inversely correlates with the activation of Akt/mTOR signaling in oral cancer cells

**Fig. 4:**
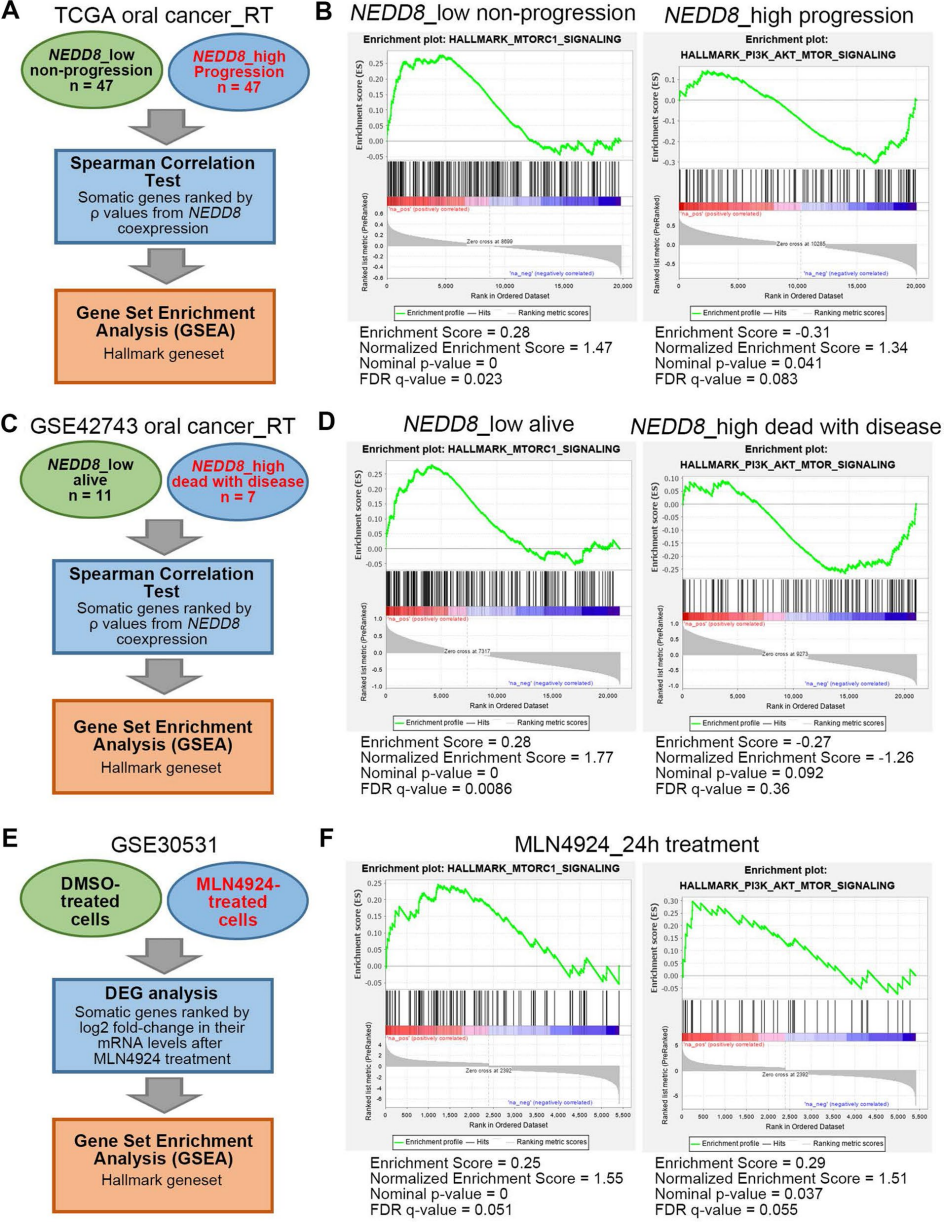
Computational simulation for the generated NEDD8 gene signature by GSEA software. **A**–**F** Flowchart of generating NEDD8 gene signatures for GSEA simulation from the indicated samples of TCGA (**A**) and GSE42743 (**C**) OSCC patients and the cell samples treated with DMSO and MLN4924 for 24 h from the GSE30531 dataset (**E**). The plots of enrichment scores among NEDD8 gene signatures, the MTORC1 gene set and the PI3K/AKT/MTOR gene set are shown in **B**, **D** and **F**. FDR denotes the false discovery rate

To ascertain the possible mechanism by which NEDD8 upregulation promotes radioresistance in oral cancer, we performed computational simulation using Gene Set Enrichment Analysis (GSEA) software against the gene signatures for NEDD8. First, we performed a Spearman correlation test against the coexpression of NEDD8 with other somatic genes in TCGA oral cancer tissues derived from radiotherapy-receiving patients who were defined as low NEDD8 expression/cancer nonprogression and high NEDD8 expression/cancer progression in Kaplan‒Meier analysis (Fig. 1E). All somatic genes were ranked by the ρ values from Spearman correlation tests and then subjected to GSEA simulation against hallmark gene sets (Fig. 4A). The data indicated that the NEDD8 gene signature derived from oral cancer tissues with low NEDD8 expression/cancer nonprogression significantly (p < 0.001) and positively correlated with the transcriptional levels of the gene set for MTORC1, whereas another NEDD8 gene signature from oral cancer tissues with high NEDD8 expression/cancer progression negatively correlated with the transcriptional levels of the gene set for the PI3K/AKT/MTOR pathway (Fig. 4B). Similar views were also found in GSEA simulation against NEDD8 gene signatures from primary tumors of radiotherapy-receiving oral cancer patients who were stratified as low NEDD8 expression/alive and high NEDD8 expression/dead with disease in Kaplan‒Meier analysis using the GSE42743 dataset (Fig. 4C and D). Moreover, we generated another NEDD8 gene signature by ranking all somatic genes based on their mRNA fold-change in A378 cells posttreatment with MLN4924 for 24 h compared to the DMSO-treated control group prior to performing GSEA simulation against hallmark gene sets (Fig. 4E). The data showed that this NEDD8 gene signature is positively correlated with the transcriptional levels of the gene set for the MTORC1 and PI3K/AKT/MTORC1 pathways (Fig. 4F).

**Fig. 5:**
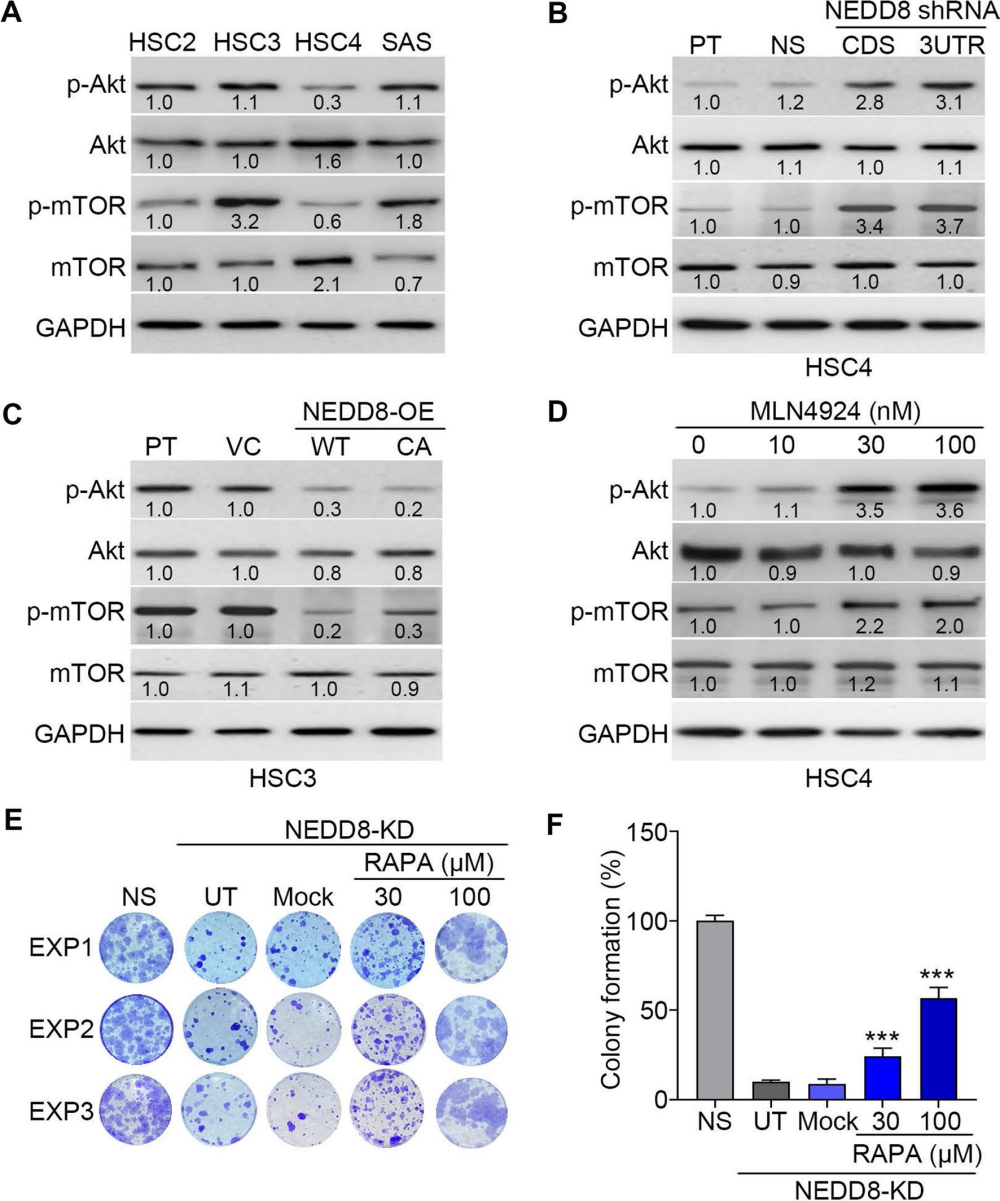
NEDD8 expression negatively regulates the activation of the Akt/mTOR signaling axis in OSCC cells. **A**–**D** Western blot analyses of the protein levels of phosphorylated Akt (p-Akt), Akt, p-mTOR, mTOR and GAPDH in the tested OSCC cell lines, HSC4 cell variants (**B**), HSC3 cell variants (**C**) and HSC4 cells treated with the indicated MLN4924 concentrations for 24 h (**D**). **E** and **F** Crystal violet staining (**E**) and histogram (**F**) for the results of three independent colony formation assays for nonsilencing control HSC4 cells and NEDD8-silenced HSC4 cells treated without (untreated, UT) or with 0.1% DMSO (Mock) and the indicated rapamycin (RAPA) concentrations for 24 h prior to treatment with irradiation at 4 Gy. The symbol “***” represents statistical significance at p < 0.001

To confirm these findings, we next dissected the endogenous protein levels of phosphorylated Akt and mTOR in OSCC cells. We found that HSC4 cells harboring a higher NEDD8 level and poorer responsiveness to irradiation treatment exhibited the lowest protein levels of phosphorylated Akt and mTOR, whereas HSC3 cells with a lower NEDD8 level and higher radiosensitivity displayed abundant expression of phosphorylated Akt and mTOR (Fig. 5A). Moreover, NEDD8 knockdown in HSC4 cells (Fig. 5B) potentiated the protein levels of phosphorylated Akt and mTOR, but NEDD8 overexpression in HSC3 cells (Fig. 5C) reduced them. Treatment with MLN4924 predominantly elevated the levels of phosphorylated Akt and mTOR in HSC4 cells (Fig. 5D). Intriguingly, the inclusion of the mTOR inhibitor rapamycin dose-dependently desensitized the NEDD8-silenced HSC4 cells to irradiation treatment (Fig. 5E and F).

### NEDD8 upregulation promotes autophagosome formation in oral cancer cells

**Fig. 6:**
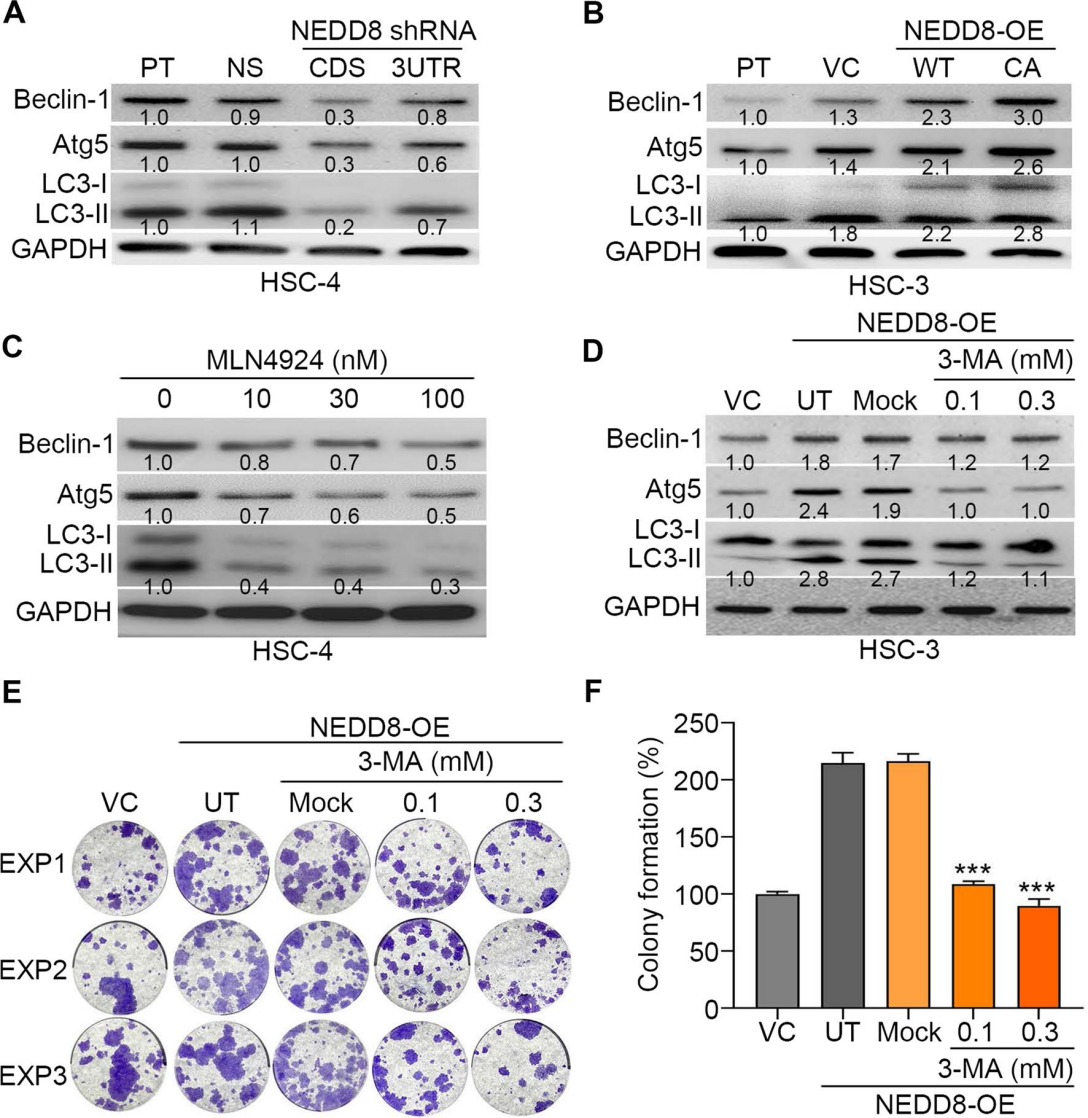
NEDD8 expression is causally associated with autophagosome formation in OSCC cells. **A–C** Western blot analyses of the protein levels of Beclin-1, Atg5, LC3-I/II and GAPDH in HSC4 cell variants (**A**), HSC3 cell variants (**B**) and HSC4 cells treated with the indicated MLN4924 concentrations for 24 h (**C**). **D**–**F** Western blot analyses for protein levels of Beclin-1, Atg5, LC3-I/II and GAPDH (**D**) and crystal violet staining (**E**)/histogram (**F**) for the results of three independent colony formation assays for vector control HSC3 cells and NEDD8-overexpressing HSC3 cells treated without (untreated, UT) or with 0.1% DMSO (Mock) and indicated 3-MA concentrations for 24 h before irradiation treatment at 4 Gy. The symbol “***” represents statistical significance at p < 0.001

Because the Akt/mTOR pathway is known to negatively regulate autophagosome formation, we next examined the correlation of NEDD8 expression with the protein levels of autophagosome components, e.g., Beclin-1, Atg5 and LC3-I/II, in oral cancer cells. We found that NEDD8 knockdown suppressed the protein expression of Beclin-1, Atg5 and LC3-I/II in HSC-4 cells (Fig. 6A), whereas NEDD8 overexpression increased the protein levels of those autophagosome components in HSC-3 cells (Fig. 6B). The pharmaceutical inhibition of NEDD8-mediated protein neddylation by MLN4924 dramatically decreased the protein levels of Beclin-1, Atg5 and LC3-I/II in HSC-4 cells (Fig. 6C). Moreover, inhibiting the initial step of autophagosome formation by 3-methyladenine suppressed the protein levels of Beclin-1, Atg5 and LC3-II (Fig. 6D) and restored the radiosensitivity of NEDD8-overexpressing HSC3 cells (Fig. 6E and F).

## Discussion

**Fig. 7:**
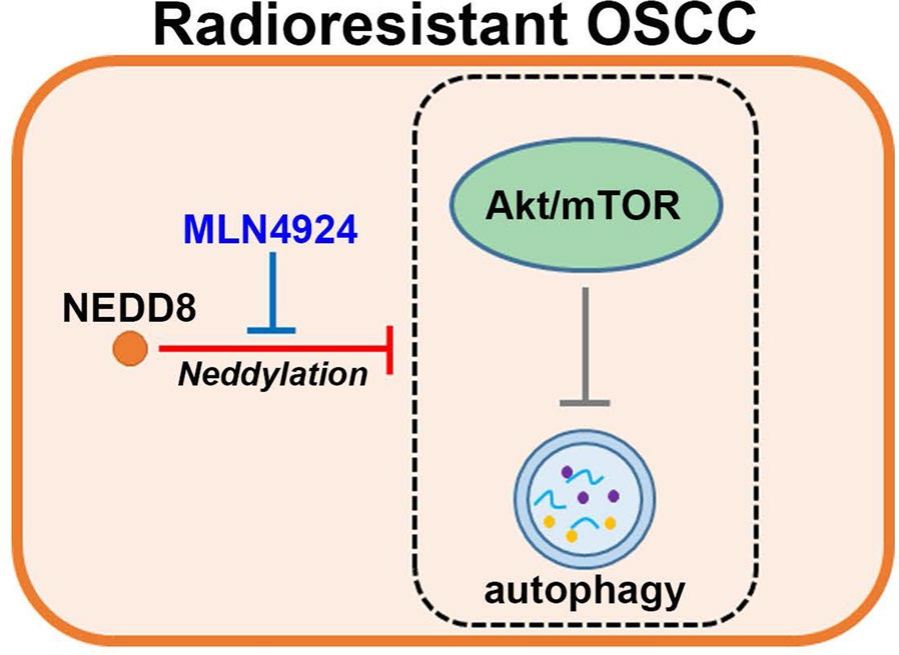
The proposed mechanism for NEDD8-conferred radioresistance in OSCC. The elevation of intracellular NEDD8-mediated protein neddylation may suppress the capacity of the Akt/mTOR signaling axis to restrain the formation of autophagy and thereby desensitize OSCC cells to irradiation treatment. The inclusion of MLN4924 suppresses protein neddylation, rescues the inhibitory function of the Akt/mTOR pathway toward autophagy formation and ultimately restores cellular sensitivity to irradiation in radioresistant OSCC

Radioresistance is a critical issue for treating OSCC patients since irradiation treatment is a main adjuvant therapy in the management of clinical OSCCs. Here, we found that NEDD8 upregulation is extensively found in primary tumors compared to normal tissues and correlates with poor responsiveness to radiotherapy in OSCC patients. Furthermore, we showed that NEDD8 knockdown dramatically sensitizes radioresistant HSC4 cells; however, NEDD8 overexpression markedly renders radiosensitive HSC3 cells more resistant to irradiation treatment. Significantly, treatment with the NEDD8 activating enzyme inhibitor MLN4924 was found to effectively suppress the therapeutic resistance of HSC4 cells to irradiation. Importantly, our results demonstrated that NEDD8 overexpression triggers autophagy activity but NEDD8 knockdown inhibits it through the negative regulation of the Akt/mTOR signaling axis in OSCC cells. These findings suggest that NEDD8 may serve as a predictive biomarker for the therapeutic effectiveness of irradiation and could be a drug target for enhancing the anticancer efficacy of radiotherapy against OSCCs (Fig. 7).

The induction of autophagy has been correlated with the mechanism for radiotherapy in several cancer types. In hepatocellular carcinoma, nuclear enriched abundant transcript 1 (NEAT1), a long noncoding RNA, conferred radioresistance through the induction of autophagy [17]. Accordingly, the induced formation of autophagy was shown to promote radioresistance in non-small cell lung cancer [18, 19], breast cancer [20], pancreatic cancer [21], esophageal cancer [22], osteosarcoma [23] and prostate cancer [24]. Inhibition of the PI3K/AKT/mTOR pathway, which is known to negatively regulate autophagy formation, appeared to promote radioresistance by inducing autophagy activity [25]; conversely, activation of the PI3K/AKT/mTOR pathway abrogated radioresistance by suppressing autophagy formation in nasopharyngeal carcinoma [26]. Recent reports have demonstrated that Wnt3a promotes radioresistance via autophagy [27], whereas the epigenetic repression of autophagy-related p62 protein overcomes radioresistance [28] in head and neck cancer. Here, we further showed that the suppression of autophagy by NEDD8 knockdown restores radiosensitivity in OSCC cells with poorer responsiveness to irradiation; in contrast, NEDD8 overexpression promotes autophagy formation and thereby renders radiosensitive OSCC cells more resistant to irradiation treatment. Therefore, NEDD8 could be a predictive biomarker for the effectiveness of radiotherapy in OSCC.

Neddylation has been closely correlated with cancer progression and poor prognosis in bladder [8] and colorectal cancers [6]. In head and neck cancer cells, the increased neddylation of p53 appeared to abolish the activity of p53 and ultimately confer radioresistance [16]. Here, we found that NEDD8 upregulation is extensively found in primary tumors compared to normal tissues derived from patients with head and neck cancer and highly correlated with poor responsiveness to radiotherapy in OSCC patients. Moreover, NEDD8 knockdown potentiated the radiosensitivity of OSCC cells but NEDD8 overexpression suppressed it, indicating the critical role of neddylation in conferring radioresistance in OSCC. Although NEDD8 expression was shown to be causally associated with autophagy activity in OSCC cells, further experiments are still needed to explore the mechanism by which the neddylation of autophagy-related molecules triggers OSCC radioresistance. Accordingly, the involvement of p53 neddylation in the mechanism of OSCC radioresistance needs to be pursued.

Autophagy is induced by several stimuli, such as high heat, nutrient starvation, oxidative stress, or hypoxia, and occurs through several steps, including initiation, nucleation and expansion. Autophagy has been considered a two-edged sword because it may promote the destruction or protection of tumor cells upon irradiation exposure and thereby lead to an improved or worsened prognosis [29]. In esophageal squamous cell carcinoma (ESCC), it has been shown that the ATPase subunit of ATP6V1C1 inhibits autophagy and enhances radiotherapy resistance [30]; in contrast, our report demonstrated that Nrf2 promotes ESCC resistance to radiotherapy through CaMKIIalpha-associated activation of autophagy [31]. In addition, a potentially functional variant of the autophagy-related gene ATG10 appeared to be associated with a worse radiotherapy efficacy in patients with nasopharyngeal carcinoma [32]; conversely, treatment with tetrandrine was found to sensitize nasopharyngeal carcinoma cells to irradiation by inducing autophagy through inhibition of the MEK/ERK pathway [33]. This controversial role of autophagy in modulating cellular radiosensitivity was attributed to the activation of its upstream regulatory pathways, including PI3K/Akt/mTOR, mitogen-activated protein kinases, and the unfolded protein response, in irradiation-treated cancer cells [29]. However, the involvement of these upstream signaling pathways in the autophagy-related radiotherapeutic response in OSCC needs to be further investigated.

Since neddylation is frequently associated with malignant evolution in cancers, targeting the NEDD8 conjugation pathway through the inhibition of its activating enzyme by MLN4924 was developed as a new anticancer strategy [9, 34, 35]. A phase Ib study demonstrated that the combination of MLN4924 with carboplatin and paclitaxel is tolerable without cumulative toxicity in patients with advanced solid tumors [36]. Radiosensitization by MLN4924 has been reported in hormone-resistant prostate cancer cells [14] and pancreatic cancer cells [15]. In addition, treatment with MLN4924 induced cell apoptosis in chronic lymphocytic leukemia B cells [11] and head and neck cancer cells [10]. Here, we found that pretreatment with MLN4924 dose-dependently potentiated the effectiveness of irradiation in irradiation-insensitive HSC4 cells. However, in contrast to a previous report [12, 13], our results demonstrated that MLN4924 enhances radiosensitivity by mitigating autophagy in OSCC cells. Although further experiments are needed to clarify this controversial consequence, the induction of autophagy by promoting the PI3K-Akt-mTOR pathway is commonly found in radioresistant OSCC [29]. In addition, the radiosensitization efficacy of MNL4924 still needs to be further tested in an OSCC mouse model prior to undergoing clinical trials with OSCC patients who do not respond to radiotherapy. Understanding other microenvironmental factors that activate the purinergic P2Y2 receptor or epidermal growth factor receptor [37] to modulate the activity of the PI3K/Akt/mTOR pathway and autophagy formation is also helpful for developing new strategies to combat radioresistant OSCC in future clinics.

## Conclusions

Our results not only identify NEDD8 as a predictive biomarker for the effectiveness of radiotherapy but also offer a new strategy for radiosensitization using MLN4924 and mTOR inhibitors in OSCC with NEDD8 upregulation.

## Supplementary Information

**Additional file 1.** **Figure S1:** Uncut blots for Figure 2C, 3D, 5A and 5B. **Figure S2:** Uncut blots for Figure 5C, 5D, 6A, 6B, 6C and 6D.

## Data Availability

Not applicable.

## Abbreviations

OSCC: Oral squamous cell carcinoma
NEDD8: Neuronal precursor cell-expressed developmentally downregulated protein 8
HNSCC: Head and neck squamous cell carcinoma
UBL: Ubiquitin-like protein
PTM: Post-translational modification
TCGA: The Cancer Genome Atlas
GEO: Gene Expression Omnibus
JCRB: Japanese Collection of Research Bioresources
DMEM: Dulbecco's modified Eagle's medium
PCR: Polymerase chain reaction
GSEA: Gene Set Enrichment Analysis
NEAT1: Nuclear enriched abundant transcript 1
ESCC: Esophageal squamous cell carcinoma
