# Supplementary material for: NEDD8 promotes radioresistance via triggering autophagy formation and serves as a novel prognostic marker in oral squamous cell carcinoma
Source: Cancer Cell Int. 2023 Mar 8;23:41. doi: 10.1186/s12935-023-02883-0 (PMC9993556; doi:10.1186/s12935-023-02883-0)
Supplement: Supplementary file 1 — Additional file 1. Figure S1: Uncut blots for Figure 2C, 3D, 5A and 5B. Figure S2: Uncut blots for Figure 5C, 5D, 6A, 6B, 6C and 6D. [file 12935_2023_2883_MOESM1_ESM.pdf]

Figure 2C

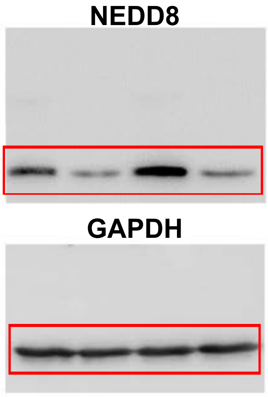

Figure 3C

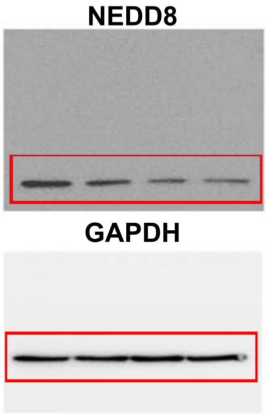

Figure 3D

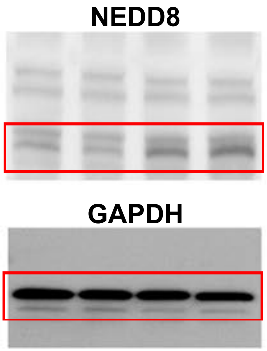

Figure 5A

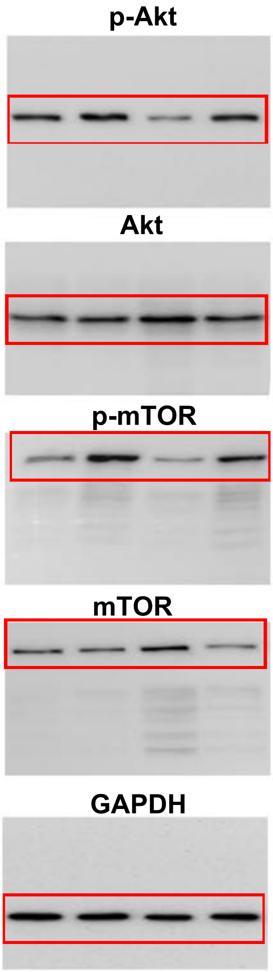

Figure 5B

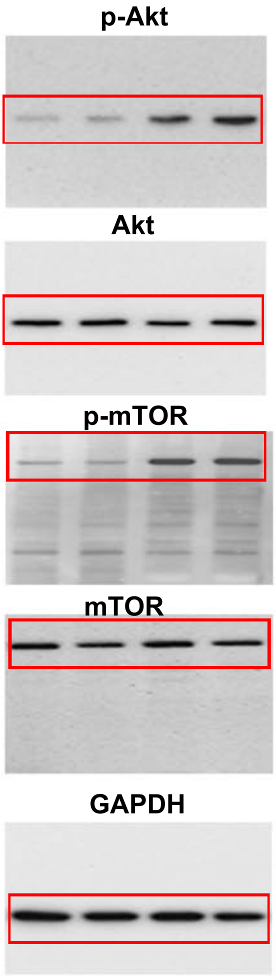

Figure S1

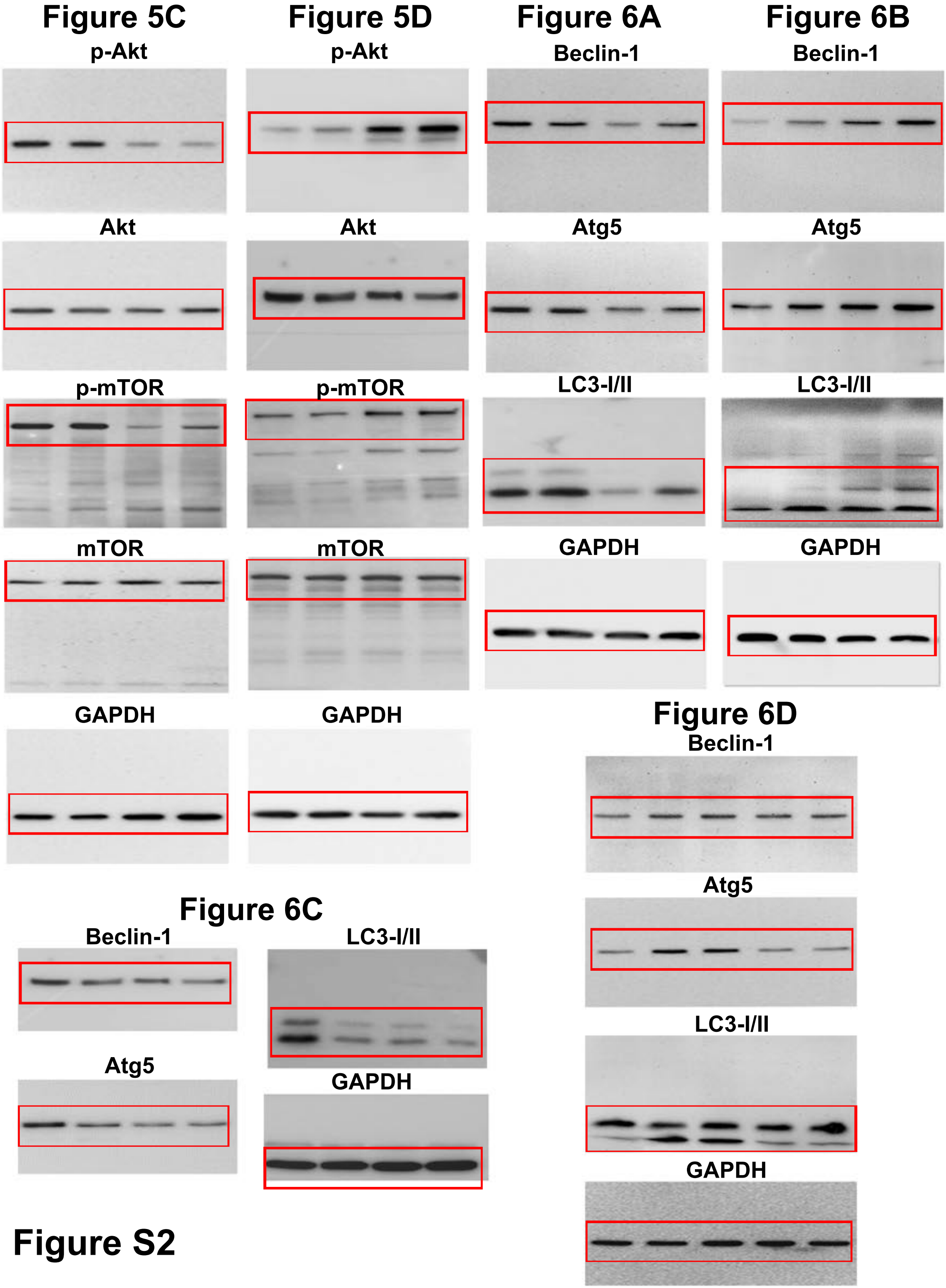
Figure 5C
p-Akt
Akt
p-mTOR
mTOR
GAPDH
Figure 5D
p-Akt
Akt
p-mTOR
mTOR
GAPDH
Figure 6A
Beclin-1
Atg5
LC3-I/II
GAPDH
Figure 6B
Beclin-1
Atg5
LC3-I/II
GAPDH
Figure 6C
Beclin-1
Atg5
LC3-I/II
GAPDH
Figure 6D
Beclin-1
Atg5
LC3-I/II
GAPDH

Figure S2
